# The lncRNAs at X Chromosome Inactivation Center: Not Just a Matter of Sex Dosage Compensation

**DOI:** 10.3390/ijms23020611

**Published:** 2022-01-06

**Authors:** Chiara Siniscalchi, Armando Di Palo, Aniello Russo, Nicoletta Potenza

**Affiliations:** Department of Environmental, Biological, Pharmaceutical Sciences and Technologies, University of Campania “Luigi Vanvitelli”, 81100 Caserta, Italy; chiara.siniscalchi@unicampania.it (C.S.); armando.dipalo@unicampania.it (A.D.P.); aniello.russo@unicampania.it (A.R.)

**Keywords:** ceRNET, microRNA, lncRNA, X chromosome inactivation, Turner syndrome, Klinefelter syndrome

## Abstract

Non-coding RNAs (ncRNAs) constitute the majority of the transcriptome, as the result of pervasive transcription of the mammalian genome. Different RNA species, such as lncRNAs, miRNAs, circRNA, mRNAs, engage in regulatory networks based on their reciprocal interactions, often in a competitive manner, in a way denominated “competing endogenous RNA (ceRNA) networks” (“ceRNET”): miRNAs and other ncRNAs modulate each other, since miRNAs can regulate the expression of lncRNAs, which in turn regulate miRNAs, titrating their availability and thus competing with the binding to other RNA targets. The unbalancing of any network component can derail the entire regulatory circuit acting as a driving force for human diseases, thus assigning “new” functions to “old” molecules. This is the case of XIST, the lncRNA characterized in the early 1990s and well known as the essential molecule for X chromosome inactivation in mammalian females, thus preventing an imbalance of X-linked gene expression between females and males. Currently, literature concerning XIST biology is becoming dominated by miRNA associations and they are also gaining prominence for other lncRNAs produced by the X-inactivation center. This review discusses the available literature to explore possible novel functions related to ceRNA activity of lncRNAs produced by the X-inactivation center, beyond their role in dosage compensation, with prospective implications for emerging gender-biased functions and pathological mechanisms.

## 1. Introduction

Non-coding RNAs (ncRNAs) constitute the largest class of the transcriptome, as the result of pervasive transcription of the mammalian genome, of which less than 2% encodes proteins [1]. Although the coding-independent functions of some ncRNAs, such as X-inactive transcript (XIST), were characterized in the early 1990s, the existence and biological relevance of the vast majority of ncRNAs were only gradually recognized a decade later, when advances in high-throughput sequencing technologies shed light on a plethora of RNA species, thus attracting tremendous interest in the field. It has become increasingly clear that ncRNAs are far from being “evolutionary junk”, and comprehension of their precious contribution to higher eukaryotes’ complexity is still at the very beginning.

Besides ribosomal RNA, ncRNA molecules can be broadly classified according to their size threshold: small or short ncRNAs, from a few to 200 nt, and long ncRNA (lncRNAs), longer than 200 nt, with a size up to several kilobases (up to 100 kb) [2].

Short ncRNAs comprise: transfer RNA (tRNA), involved in translation of mRNA; small nuclear RNA (snRNA) and small nucleolar RNA (snoRNA), involved in splicing and in ribosomal RNA modification, respectively; Piwi-interacting RNA (piRNA), involved in transposon repression; microRNA (miRNA), the most studied group of small ncRNAs, with a size of approximately 20 nt. miRNAs are post-transcriptional regulators of gene expression by binding to target transcripts, thus affecting their translation and/or stability [3]. Mammalian miRNAs are predicted to regulate up to 50% of all protein-coding genes, wherein each miRNA can bind different mRNAs and each mRNA can be targeted by various miRNAs, giving rise to complex regulatory networks that play key roles in almost all physiological pathways, but also in the pathogenesis of several diseases, especially cancer, where they can act as oncogenes (oncomiRs) or tumor suppressors [3,4,5].

lncRNAs constitute the largest class of ncRNAs in the mammalian genome and can be further classified into subclasses: long intergenic ncRNA (lincRNA), transcribed from intergenic regions, endowed with their own regulatory elements; sense lncRNAs, transcribed in the same direction as a coding gene, overlapping one or more exons or embedded in one of the introns (intronic lncRNA); antisense RNA (asRNA), transcribed as antisense strands compared to an overlapping known gene; pseudogenes, a version of coding genes that lost their protein-coding capacity due to mutations; circular RNA (circRNA), arising from backsplicing events of protein-coding transcripts that form covalently closed continuous loops; enhancer RNA (eRNA), deriving from enhancers endowed with enhancer-like functions [6].

lncRNAs and coding transcript biogenesis share various features: transcription, generally by RNA polymerase II, 5′-capping and 3′-polyadenylation modifications and also splicing. Additionally, miRNAs are transcribed by RNA polymerase II as long primary transcripts, and then subjected to a maturation compartmentalized between the nucleus and cytoplasm [3]. In contrast to mRNAs and miRNAs, largely operative in the cytoplasm, lncRNAs can be retained in the nucleus [7]. Unlike protein-coding transcripts and miRNAs sequences, lncRNAs are evolutionarily less conserved, however, conservation may be found in secondary structures that enable them to bind to proteins, DNA and other RNA molecules [8,9]. In the nucleus, lncRNAs can function as gene expression regulators by interacting with DNA, chromatin modifying complexes and/or various transcriptional regulators; in the cytoplasm, by binding proteins and RNA molecules, lncRNAs can still regulate gene expression at the post-transcriptional level by sponging miRNAs, regulating mRNA degradation and translation, and short open reading frames can even serve as templates for the synthesis of “micropeptides” [2,10]. Like miRNAs, lncRNA can also be secreted and found in the extracellular space [11]. Finally, lncRNA expression is finely regulated in physiological conditions, and their dysregulation contributes to pathogenesis of several diseases [7,12,13].

It is becoming increasingly clear that ncRNAs engage in regulatory networks based on interactions among the different RNA species, often in a competitive manner, in a way denominated “competing endogenous RNA (ceRNA) networks”, abbreviated as “ceRNET”; miRNAs and other ncRNAs modulate each other, since miRNAs can regulate the expression of lncRNAs, which in turn regulate miRNAs, titrating their availability and thus competing with the binding to other RNA targets [14,15,16]. In this scenario, coding transcripts themselves have a regulatory potential beyond their coding ability, when they compete for binding to shared miRNAs [16,17]. Indeed, transcripts from pseudogenes should also not be considered “evolutionary relics”, but can regulate expression of their related gene by competitively binding shared miRNAs [18]. In a physiological state, an optimal crosstalk occurs, since the shared pool of miRNAs is sufficient to target repression, whereas the unbalancing of any network component can derail the entire regulatory circuit acting as a driving force for human diseases. In this new perspective of RNA crosstalk, behind the conventional miRNA unidirectional regulation of target transcripts, different studies have highlighted additional roles of well-known lncRNAs as miRNA decoys with the effect of preventing their inhibitory binding to specific mRNA targets. Furthermore, it is also possible for lncRNAs and miRNAs with different preferential subcellular localizations to participate in the same ceRNA networks, even by shuttling between different compartments upon specific physiological or pathological conditions [16,19,20]. This is the case of XIST, the lncRNA well known for its role as a major and indispensable effector of X chromosome inactivation in mammalian females that prevents an imbalance of X-linked gene expression between females and males (see next paragraph); despite XIST’s preferential nuclear localization, and canonical miRNA function in the cytosol, many works revealed that XIST could also function as a ceRNA by sponging different miRNAs and thus de-repressing different protein-coding RNAs. Actually, literature concerning XIST biology is becoming dominated by miRNA associations and sponging activity [19], and this is also becoming true for other lncRNAs, also produced by the X-inactivation center (Xic) and involved in fine-tuning XIST expression. However, implications of those ceRNETs involving sex-specific lncRNAs in emerging gender-biased functions and pathological mechanisms are still poorly explored. With this perspective in mind, this review wants to explore possible new functions related to ceRNA activity of lncRNAs produced by the X-inactivation center, beyond their well-established role in the inactivation of the X chromosome of mammalian females.

## 2. Dosage Compensation and X-Inactivation Center

The presence of two X chromosomes in females and only one X chromosome in males requires mechanisms to equalize gene dosage between sexes and relative to the other chromosomes (autosomes), thus avoiding a potentially lethal double dose of genes localized on the X chromosome [21]. In mammals, dosage compensation between females and males is achieved through the process named X chromosome inactivation (XCI), i.e., the silencing of one of the two X chromosomes in females, a specialized form of heterochromatin formation established during early development and maintained through cell division and the adult body. This process, proposed for the first time by Lyon, ensures that the number of active X chromosomes in male and female cells is equalized with only one X chromosome being active (Xa) [21]. Different species employ different strategies for chromosome-wide inactivation with regard to the parental origin of the inactive X chromosome (Xi), being imprinted XCI in marsupials and monotremes, and random XCI in eutherians. The developmental timing and mode of chromosome selection for inactivation also varies. As an example, in mice, from the 8-cell to the morula stage, the paternal X is progressively inactivated, and then reactivated in the inner cell mass at the blastocyst stage, followed by random XCI, whereby both X chromosomes have the same probability of being inactivated and then epigenetically maintained; in humans, the two X chromosomes are active in the early cleavage stage, random XCI occurs at the morula stage and it persists in blastocysts in embryonic and extra-embryonic lineages [22]. However, a common feature of gene dosage compensation systems is their dependence on lncRNAs. In particular, in eutherians, the lncRNA XIST is the master regulator of the XCI process, whose genomic organization and function is widely conserved. XIST is transcribed by the X-inactivation center (XIC), then it coats and propagates along the future Xi, packaging it into transcriptional inactive heterochromatin through interaction with several protein partners [23].

Beyond the intricate molecular mechanisms underlying the XCI, it has also been shown that some genes escape X inactivation and are expressed from both the active and inactive X chromosome. Such genes are potential contributors to sexually dimorphic traits, to phenotypic variability among females heterozygous for X-linked conditions and to clinical abnormalities in patients with abnormal X chromosomes. Up to 15% of X-linked genes escape inactivation with large variability in their number and tissue distribution within a given individual and between individuals; the escape from silencing or skewed XCI allows the expression of some genes by both X chromosomes in females; in addition, skewed XCI may be also a consequence of early embryonic cell death [24,25,26].

**Figure 1:**
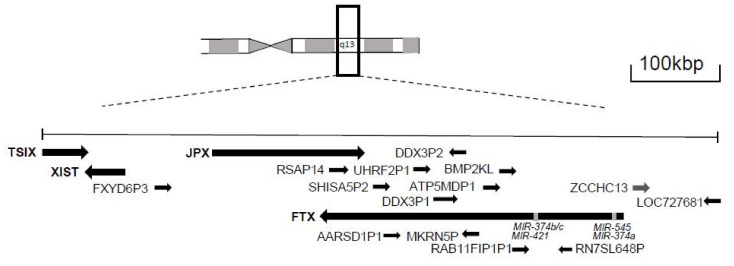
Human X chromosome inactivation center. Representation of a portion of the X chromosome, with zoomed-in view of the region that controls XCI. XIC harbors many lncRNA genes and one protein-coding gene, ZCCHC13 (gray arrow); a few other protein-coding genes are located upstream of TSIX. The most well-characterized lncRNAs are indicated in bold font and with thicker arrows; all the others are inferred pseudogenes and indicated by thinner arrows. The direction of the arrows indicates the direction of transcription. Positions of the miRNA clusters on FTX are also indicated. Data were retrieved from Ensembl and NCBI databases.

The human XIC is the X-linked minimal genetic region that is necessary and sufficient to initiate XCI; it spans approximately a 1 Mb region in Xq13 [27]; it produces few coding transcripts, various uncharacterized inferred pseudogene transcripts and it is particularly enriched in genes producing lncRNAs, with some of them better characterized (see next paragraphs) and acting as activators or repressors engaged in a complex interplay regulating the monoallelic expression of XIST (Figure 1). Tridimensionally, the XIC is physically and spatially organized in two topologically associated domains (TADs): the TSIX TAD that includes a repressor of XCI, and the XIST TAD encompassing XCI activators. Despite the overall XIC gene synteny, order and orientation conservation, the human XIC locus is considerably expanded compared to the mouse ortholog region, and comprises different “actors’’ [28]; some differences can be observed in the organization and structure of the X-inactivation center, with a lesser degree of conservation for the TSIX TAD and dynamics of XIST expression during early embryogenesis. The generally poor conservation of the sequence of various lncRNAs implicated in a conserved process such as XCI suggests that rapidly evolving lncRNAs might favor adaptation to species-specific developmental/environmental constraints and acquire diversified functions. It is paradigmatic of the versatility of lncRNAs’ structure and function that an essential phenomenon may follow different routes, just sustained by ncRNAs.

Below there is a summary of the most well-characterized lncRNAs and their contribution to the mechanism of XCI.

### 2.1. XIST

XIST was one of the first long non-coding RNAs to be discovered in the early 1990s, first in humans and soon after in mice [23], being 17 kbp long in mice (Xist) and 19 kbp long in humans (XIST), transcribed by polymerase II, polyadenylated and retained in the nucleus [29]. Xist/XIST is the master regulator of XCI, transcribed exclusively from the (Xi), and then spreading along the X chromosome from which it was transcribed [30].

XIST is necessary and sufficient for starting the chromosome-wide gene silencing by cis-coating the future inactive X chromosome (Xi), mediating a cascade of epigenetic modifications and structural reshaping of the heterochromatin, culminating in the Xi becoming condensed in the so-called Barr body, which is maintained through multiple rounds of cell division [27,31,32]. Imaging studies revealed that X-linked genes, initially located at the periphery of the Xist RNA cloud, adopt a more internal position when silenced [33,34]. Exact mechanisms and factors whereby Xist initiates X inactivation are still under investigation; complex interactions occur between epigenetic and genomic features, such as genomic distance from the Xist locus, gene density and proximity to long interspersed nuclear elements (LINEs) that act as waystations to enhance Xist RNA coating throughout the Xi 3D space [35].

Despite the generally weak sequence conservation of Xist among eutherian mammalian, conservation is observed for the global gene structure and especially for the blocks of tandem repeats named A–F-repeats along its eight exons, cooperatively enabling Xist interaction with transcriptional factors, scaffold proteins and chromatin-modifying proteins, and thus mediating silencing and Xist localization on Xi [22,36,37,38,39]. In particular, the A-repeat, consisting of 7.5 copies of a 26 nt core sequence at the 5′ end of Xist RNA, is not involved in the coating mechanism, but in triggering gene silencing by recruiting the transcriptional repressor SPEN [40,41,42]; the A-repeat directly binds Polycomb repressive complex 2 (PRC2) components, thus mediating PRC2 recruitment to the Xi. The B-repeat, together with a short part of the C-repeat, is crucial for Xist spreading and for the recruitment of the Polycomb repressive complex 1 (PRC1), that could in turn recruit PRC2 [43,44,45]. The C-repeat, along with a ‘’nucleation center’’ for spreading across the Xi at the F-repeat, is involved in the interaction with the transcription factor Ying Yang 1 (YY1), that acts as an anchoring point to bridge between Xist RNA and the Xist gene [46] at an initial phase of X inactivation. Another interactor involved in Xist localization and X-linked gene silencing is the heterogeneous nuclear ribonucleoprotein U (hnRNP U), which interacts with Xist RNA by binding to exon 1 and 7 of both human and mouse XIST/Xist RNA [23] and it acts as a bridge between the matrix/scaffold-attached region in the Xi and Xist to facilitate spreading of the silencing machinery such as PRC2.

Of fundamental importance in the complex scenario of XCI is the recruitment of the Polycomb repressive complexes PRC1 and PRC2 by Xist RNA, indeed representing an important paradigm for chromatin regulation by long non-coding RNAs [47,48]. Then, other mechanisms are believed to act synergistically to maintain the inactive state, such as histone modifications, including loss of active histone marks (H3 and H4 acetylation, H3K4 methylation) and gain of repressive histone modifications (H3K9, H3K27 and H4K20 methylation), enrichment in the histone variant macroH2A and DNA methylation of the CpG island at the promoters of the X-linked genes [49].

A complex picture comes out from the abovementioned studies, whereby XIST acts as a trigger for Xi, and its structural versatility works as a scaffold for the recruitment of so many complexes to establish and maintain the inactive state.

### 2.2. JPX

Just proximal to XIST (JPX) is a long non-coding RNA transcribed from a gene within the X-inactivation center located ~10 kb upstream of XIST and expressed in the antisense direction. It lacks open reading frames but is relatively conserved in its 5′ exons. JPX was hinted as a key regulator in the positive arm of Xist regulation although the exact mechanisms of action need to be explored further [50]. The Jpx gene, also known as Enox, is less conserved than Ftx, the other positive regulator of Xist, showing a high identity with human JPX only at the level of one exon [28].

Experiments conducted on mouse embryonic stem cells (ESCs) revealed that Jpx escapes X chromosome inactivation, is upregulated during X-inactivation and it is required for the proper expression of Xist. Indeed, time-course measurements of both Jpx and Xist displayed increased Jpx RNA levels; this upregulation occurred in both XX and XY cells, however, whereas Xist induction followed Jpx regulation in female cells, Xist remained suppressed in male cells. Of note is the fact that the deletion of a single Jpx allele in female cells is sufficient to prevent X chromosome inactivation, thus reinforcing its role as an indispensable element in orchestrating the process. Moreover, through expressing Jpx from an autosomal transgene into ΔJpx/+ cells it was shown that Jpx levels were restored, implying that Jpx must therefore be able to act predominantly in trans, showing only a mild cis preference [50]. Based on overexpression experiments conducted on mouse ESCs, Sun and colleagues demonstrated the existence of a correlation between Jpx levels and Xist induction, indeed, during the different stages of differentiation, Xist showed a dose-dependent response to Jpx expression. One of the most interesting features of the regulation exerted by Jpx regards a sort of mechanism of molecular titration between Jpx and the RNA-binding protein CTCF that dictate Xist induction in opposite manners. Specifically, CTCF binds the Xist promoter P2 in mice and correlates with Xist repression. These findings were supported by the demonstration that CTCF overexpression blunted Xist upregulation and implied that CTCF is a blocking factor for Xist, acting in an opposite manner to Jpx. In addition, the fact that CTCF persisted at Xist P2 when Jpx was deficient hinted that Jpx and CTCF might be antagonistically linked. In the same study, in fact, new important evidence emerged: Jpx activates the Xist promoter by removing CTCF protein from the Xist promoter on the Xi [51]. A recent study developed a novel transgenic mouse system to demonstrate the regulatory mechanisms of lncRNA Jpx: the authors observed a dose-dependent relationship between Jpx copy number and Xist expression in mice, and that transgenic Jpx can activate the endogenous Xist in trans, suggesting that Jpx is sufficient to activate Xist expression in vivo; then, the authors proposed that Jpx is also able to act in cis [52], as previously found by others [53,54]. However, whether Jpx effectively acts in cis or in trans is still a controversy and needs to be further clarified.

### 2.3. FTX

Five prime to Xist (FTX) is another gene located upstream of XIST, within the XIC. It produces a long non-coding RNA that positively regulates the expression of XIST. Comparative analyses of ESTs revealed that the murine Ftx gene is composed of 15 exons spanning about 63 kbp, while human FTX comprises 12 exons spanning about 330 kbp and both mouse and human FTX genes give rise to various transcripts that originate from different combinations of alternative promoter usage and splicing. Strikingly, the Ftx gene harbors the conserved miRNA cluster miR-374b and 421 (miR-374 and miR-421 in mice) and only in humans is there a second cluster composed of miR-374a and miR-545, localized in intron b. Sequence analyses in humans revealed that miR-374a and miR-545 show similarities to miR-374b and miR-421, suggesting that they arise from duplication.

By means of mouse ES cells, it has been possible to demonstrate that Ftx is specifically upregulated during female cell differentiation at the onset of XCI, reminiscent of Xist expression, and the deletion of the promoter region of Ftx leads to local alterations in the chromatin structure. Strikingly, Xist is significantly downregulated in the absence of Ftx, suggesting that Ftx acts as an activator [55].

Additional evidence, regarding the involvement of Ftx for proper Xist expression dynamics and for efficient XCI progression, arose from a recent study in which several experiments at different levels were carried out in mutant mouse ESCs that were engineered by CRISPR-Cas/9, in order to delete a 9 kb region including the three putative promoters of Ftx. The data revealed that Ftx influences the efficiency of Xist activation and indicate that transcription from Ftx promoters is required for proper Xist expression in cis, independently of Ftx lncRNA transcripts produced from the locus or of Ftx-embedded miRs [56]. Moreover, previous observations that Ftx escapes XCI [55] and that it is required in cis for Xist accumulation were confirmed [56].

In this context, Ftx represents one important component of the delicate balance of activators and repressors in the multiplicity of layers operating in the organization of XCI and driving robust Xist upregulation.

### 2.4. TSIX

The Tsix gene expresses a non-coding transcript across the 3’ end and antisense to XIST, named “Tsix”, that is, Xist spelled in reverse order; conversely to Jpx and Ftx, it plays an antagonistic role in Xist expression. Tsix RNA is dynamically regulated during X inactivation: before X inactivation, Tsix is biallelically expressed but becomes monoallelically expressed at the onset of X inactivation, marking only the future active X and therefore raising the possibility that Tsix blocks Xist accumulation. By carrying out targeted deletion in female and male mouse cells of the 5′ CpG-rich domain of Tsix, it was demonstrated that Tsix encodes a single 40 kbp antisense transcript rather than multiple smaller RNAs and it is required for the random nature of X chromosome choice. Moreover, Tsix acts exclusively in cis and marks the future active X chromosome by blocking Xist accumulation [57]. Tsix was initially described as a 40 kb RNA encoded by a single exon, but later studies showed that it is, at least in part, subjected to splicing [58]. Even though Tsix plays an important role in mouse X inactivation, it is less conserved in humans at the sequence level [28].

Human TSIX produces a >30 kb transcript that is expressed only in cells of fetal origin; it is expressed from human XIC transgenes in mouse embryonic stem cells and from human embryoid-body-derived cells, but not from human adult somatic cells. Differences in the structure of human and murine genes suggest that human TSIX was truncated during evolution, and these differences could explain the fact that X inactivation is not imprinted in human placenta, thus raising questions about the role of TSIX in random X inactivation [59]. A later study from the same authors shed light on other interesting features regarding the differences between the two species; specifically using RNA FISH for cellular localization of transcripts in human fetal cells, they showed that human TSIX antisense transcripts were unable to repress XIST. Indeed, TSIX was transcribed only from the inactive X chromosome and was coexpressed with XIST, implying that the repression of Xist by mouse Tsix has no counterpart in humans, and TSIX is not the gene that protects the active X chromosome from random inactivation [60]. Regarding the role of Tsix in the chromatin modifications at the Xist locus, of note is the “dual effect” exerted by “opening” the chromatin structure along the Xist gene and “closing” it at the Xist promoter itself [61,62].

Overall, the interplay of different lncRNAs from the XIC secures the monoallelic Xist expression by acting as positive (FTX, JPX) and negative (TSIX) regulators and bringing about Xi in only one of the two X chromosomes in females.

## 3. ceRNA Activity of the lncRNAs from the XIC

### 3.1. ceRNETs Involving XIST

In recent years, literature concerning XIST biology has come to be dominated by miRNA associations. In particular, by searching “(XIST) AND (miRNA)” throughout PubMed, 257 results were retrieved that were published before 30 September 2021. A deep inspection of these articles led to the results presented in Table 1, selecting only those reporting miRNAs whose binding to XIST and the competitor transcripts was experimentally validated by RNA immunoprecipitation (RIP) and/or luciferase and/or RNA pull-down assays; then, the list of miRNAs and related mRNA targets was further processed by grouping similar “biological contexts” or similar “effects” or common components of the networks to envisage the potential overall framework. In addition, studies performed on mice and rats were also included, based on the consideration that despite some differences in nucleotide sequence, human lncRNA homologs may functionally compensate for the loss of mouse homologs [63].

The different studies mainly related the miRNA sponging activity of XIST to an oncogenic role in different types of cancer. In the case of colon cancer, the studies consistently point to an oncogenic role of XIST: it was upregulated in colon cancer tissues compared to the non-tumoral tissues and correlated to poor prognosis; it sponges miR-34a, thus preventing miRNA binding to WNT1, thus triggering the Wnt/beta-catenin pathway activation and then promoting proliferation and invasion of colon cancer cells [64]. A functionally similar axis involving XIST, miR-486-5p and neuropilin-2 has also been found to be involved in the epithelial–mesenchymal transition of colorectal cancer cells, thus participating in cancer progression [65]. Intriguingly, the XIST ceRNA activity is also involved in chemoresistance: as an example, XIST competes with serum and glucocorticoid-inducible kinase 1 (SGK1) by sponging the shared miR-124, and thus enhancing doxorubicin resistance [66]. Recently, complex lncRNA/miRNA/mRNA networks based on XIST/miR-500a-3p, miR-370-3p, miR-2467-3p, miR-512-3p/XBP have been demonstrated to promote cell proliferation and aggravated tumor growth in vivo by regulating endoplasmic reticulum stress response and cell apoptosis [67].

In the context of gastrointestinal tumors, XIST was also found to be overexpressed in gastric cancer tissues and associated with an aggressive tumor phenotype and adverse prognosis; XIST’s oncogenic power was demonstrated both in vitro and in vivo and at least partly attributed to the sponging activity of miR-101, resulting in the de-repression of the Polycomb group protein enhancer of zeste homolog 2 (EZH2) and promotion of cancer progression and metastasis [68]. Similarly, XIST upregulated Paxillin (PXN) expression by competitively binding to miR-132, and thus promoting carcinogenesis [69].

An oncogenic role of XIST mediated by its ceRNA activity has also been consistently indicated by different studies of non-small-cell lung cancer (NSCLC). In one pathway, XIST upregulation in NSLC tissues led to sequestering of miR-367 and miR-141, resulting in the de-repression of their target zinc finger E-box binding homeobox 1 (ZEB2), a transcriptional repressor of E-cadherin, and promoting cell invasion and metastasis; both in vitro and in vivo experiments link XIST to TGF-beta-induced epithelial–mesenchymal transition via miRNA crosstalk [70]. Different axes involving XIST/shared miRNA/mRNA target, and reported in Table 1, point to a role of XIST in cell proliferation, migration, invasion, EMT and chemoresistance of lung cancer, prospectively suggesting new therapeutic targets.

Similarly to ZEB2, ZEB1 from the same protein family has also been linked to an oncogenic role of XIST in another kind of tumor: in pancreatic cancer, XIST was frequently upregulated, especially in metastatic tissues, and sponges the tumor suppressor miR-429, thus acting as a ceRNA for ZEB1 that was upregulated; in particular, the axis XIST/miR-429/ZEB1 is critical for cell migration, invasion and EMT [71]. In the same pathological pathway, a role for the XIST/miR-141-5p/TGF-β2 axis has been reported [72].

Both ZEB1 and 2 have also been found to be involved in retinoblastoma, the most frequent eye malignancy in childhood, where XIST’s oncogenic role has been demonstrated in terms of promotion of proliferation, migration, invasion and EMT; again, XIST acts as a sponge for miR-101, thus resulting in the upregulation of its targets ZEB1 and ZEB2 [73]. Other works reported in Table 1 consistently indicate the ceRNA activity of XIST as a mechanism promoting retinoblastoma progression. In the context of pediatric tumors, in neuroblastoma an oncogenic role for XIST has also been determined and related to its ceRNA activity. In particular, XIST depletion repressed tumor growth in vivo and increased radiosensitivity, arrested cell cycle progression and impeded proliferation of neuroblastoma cells in vitro; mechanistically, XIST modulated L1 cell adhesion molecular (L1CAM) expression by competitively binding to miR-375 [74].

The oncogenic role of XIST has also been coherently reported by different papers studying another tumors of the nervous system: in glioma tissues and cell lines, XIST is significantly upregulated and related to poorer clinical and pathologic features and shorter survival time; its knockdown inhibits cell proliferation, migration and invasion in vitro and reduces tumor growth in vivo [75,76,77,78]. Again, the mechanism of action seems to be based on regulatory RNA networks involving XIST, miRNAs and their main targets. In one pathway, XIST participates in glioblastoma progression by enhancing glucose metabolism via sponging miR-126 and then preventing its binding to insulin receptor substrate 1 (IRS1), thus modulating the IRS1/PI3K/Akt pathway [75]. Functionally similar axes based on XIST/miR-133a/SOX4 and XIST/miR-329-3p/CREB1 networks have been found to be involved in promoting cell proliferation and metastasis [78,79].

The ceRNA activity of XIST versus miR-34a has an oncogenic effect in two different tumors, thyroid cancer and nasopharyngeal carcinoma, due to de-repression of different targets in different cell contexts, i.e., MET and E2F3, respectively [80,81].

With regard to female cancers, an oncogenic role has been attributed to XIST and associated with ceRNA activity. XIST was upregulated in cervical cancer tissues and cell lines and predicted unfavorable prognosis of patients. In vitro and in vivo experiments showed that it contributes to progression of cancer through different axes, i.e., miR-200a/Fus, miR-140-5p/ORC1, miR-889-3p/SIX1, that could be collectively explored for development of new therapeutic methods [82,83,84]. In ovarian cancer, XIST, miR-140-5p and the transcriptional factor FOXP3 engage a ceRNET, driving ovarian cancer cell progression: XIST was found upregulated in cancer tissues and correlated to poor prognosis of patients; conversely, miR-149-3p was found downregulated with the consequent upregulation of its target, FOXP3; XIST knock-down elevated miR-149-3p to suppress migration and invasion of ovarian cancer cells, and restricted tumor growth in vivo [85]. A similar regulatory network has been identified, whereby XIST regulated proliferation, invasion and migration via the miR-335/BCL2L2 axis [86].

An oncogenic role of XIST has been coherently indicated for other tumors, such as laryngeal squamous cell carcinoma, esophageal cancer and osteosarcoma (Table 1), whereas contrasting results have been published for hepatocellular carcinoma (HCC), where a tumor suppressor role has mainly been attributed. In particular, XIST could inhibit HCC cell proliferation and metastasis by sponging miR-92b, whose oncogenic role was demonstrated in vitro and in vivo and is at least partly due to the regulation of its target Smad7 [87]. Then, functionally similar networks were described involving XIST/miR-155-5p/SOX6, PTEN and XIST/miR-497-59/PDCD4; accordingly, XIST overexpression inhibited tumor growth in vivo [88,89]. These findings, together with the notion of a prevailing expression of XIST in females, may suggest an additional interpretation of the gender disparity of HCC occurrence (incidence rate 2–3 times higher in males than in females), to date related to sex hormones and cytokines [90,91].

Other phenomena linked to ceRNA activity of XIST are listed in Table 1. Among them, some papers accordingly indicate a proinflammatory role in different pathological pathways (Table 1) [92,93,94], including osteoarthritis [95,96]. XIST is upregulated in human osteoarthritis specimens and contributes to the pathogenesis by engaging different regulatory networks: it competitively binds to miR-376c-5p to increase the expression of osteopontin (OPN), exacerbating the inflammatory microenvironment and modulating the influence of proinflammatory M1 macrophages on chondrocyte apoptosis [95]; it binds to miR-211, thus increasing CXCR4, a major contributor to chondrocyte apoptosis [97]; it promotes extracellular matrix degradation by sponging miR-1277-5p, thus de-repressing matrix metalloproteinase 13 (MMP-13) and ADAM metallopeptidase with thrombospondin type 1 motif 5 (ADAMTS5) [98]; finally, two additional regulatory axes have been recently reported, miR-130/STAT3 and miR-149-5p/DNMT3A, where XIST was also confirmed to promote pathogenesis [96,99].

In the context of cardiovascular diseases, XIST was found upregulated in coronary heart disease tissues and post-myocardial infarction cells, where it suppressed cell proliferation and promoted apoptosis, at least partly by XIST/miR-130-3p/PDE4D [100]. Similar effects were observed in myocardial cell apoptosis in acute myocardial infarction model rats through XIST/miR-449/Notch1 [101]. Other works also point to a role of XIST in cardiovascular homeostasis, although in different model systems, and with some contrasting results, probably due to different disease models, and thus requiring further investigation [102,103].

The complex picture emerging from the data reported in Table 1 indicates that XIST is implicated in many pathways other than dosage compensation, especially in cancer, where it is often upregulated and acts as an oncogene by derailing the RNA networks involved in the control of cell proliferation, invasion, EMT and chemoresistance. However, much work is still required to envisage a common scenario, also complicated by the fact that a lncRNA may carry various binding sites for interaction with different miRNAs, and combination/coexpression of miRNAs and potential targets in different cell contexts may drive different, sometimes contrasting, effects. As an example, the interaction between XIST and miR-34a has been validated in different cell contexts, and implicated in nasopharyngeal carcinoma and colon and thyroid cancer, by networking with different mRNA targets, i.e., E2F3, WNT1 and MET, respectively [65,81,82], as shown in Table 1. Vice versa, the same mRNAs in combination with different XIST/miRNA axes can have relevance in different pathological contexts; as an example, the ZEB1/ZEB2 family has been implicated in NSCLC, pancreatic cancer, retinoblastoma and HCC in combination with different XIST/miRNA axes [71,72,74,104], as shown in Table 1.

**Table 1:** ceRNETs involving lncRNAs from XIC.

Competing Endogenous RNAs					
lncRNAs	mRNAs	Shared miRNAs	Context	Effect	Ref.
XIST	WNT1	miR-34a	Colon cancer	Oncogenic role	[64]
	neuropilin-2	miR-486-5p		Oncogenic role	[65]
	XBP-1	miR-500a-3p,miR-370-3p, miR-2467-3p, miR-512-3p		Oncogenic role	[67]
	PAX5	miR-338-3p		Oncogenic role	[105]
	HIF-1A	miR-93-5p			[106]
	SGK1	miR-124		Doxorubicin resistance	[66]
	ROR1	miR-30a-5p		Chemoresistance	[107]
	WEE1	miR-125b-2-3p		Oncogenic role and chemoresistance	[108]
	EZH2	miR-101	Gastric cancer	Oncogenic role	[68]
	PXN	miR-132		Oncogenic role	[69]
	JAK2	miR-337		Oncogenic role	[109]
	ZEB2	miR-367, miR-141		Oncogenic role	[70]
	Notch-1	miR-137	NSCLC	Oncogenic role	[110]
	SOD2	miR-335		Oncogenic role	[111]
	RING1	miR-744		Oncogenic role	[112]
	PAX6	miR-142-5p		Oncogenic role	[113]
	ATG7	miR-17		Cisplatin resistance	[114]
	MDM2	miR-363-3p	LUAD	Oncogenic role	[115]
	ZEB1	miR-429	Pancreatic cancer	Oncogenic role	[71]
	TGF-β2	miR-141-3p		Oncogenic role	[72]
	Notch1	miR-137		Oncogenic role	[116]
	ZEB1, ZEB2	miR-101	Retinoblastoma	Oncogenic role	[73]
	STAT3	miR-124		Oncogenic role	[117]
	SOX4	miR-140-5p		Oncogenic role	[118]
	BDNF	miR-191-5p		Oncogenic role	[119]
	STX17	miR-361-3p		Oncogenic role	[120]
	L1CAM	miR-375	Neuroblastoma	Oncogenic role	[74]
	bFGF (FGF2)	miR-424-5p	Pituitary neuroendocrine tumor	Oncogenic role	[121]
	IRS1	miR-126	Glioma	Oncogenic role	[75]
	SOX4	miR-133a		Oncogenic role	[79]
	CREB1	miR-329-3p		Oncogenic role	[78]
	ROCK1	miR-448		Oncogenic role	[122]
	SP1, MGMT	miR-29c		Temozolomide chemoresistance	[77]
	MET	miR-34a	Thyroid cancer	Oncogenic role	[80]
	CLDN1	miR-101-3p		Oncogenic role	[123]
	E2F3	miR-34a-5p	Nasopharyngeal carcinoma	Oncogenic role	[81]
	ADAM17	miR-148a-3p		Oncogenic role	[124]
	NEK5	miR-381-3p		Oncogenic role	[125]
	RECK	miR-30b		Oncogenic role	[126]
	EZH2	miR-124	Laryngeal squamous cell carcinoma	Oncogenic role	[127]
	IRS1	miR-144		Oncogenic role	[128]
	TRIB2	miR-125b-5p		Oncogenic role	[129]
	CDK6	miR-494	Esophageal cancer	Oncogenic role	[130]
	mTOR	miR-375-3p	Osteosarcoma	Oncogenic role	[131]
	RAB16	miR-758		Oncogenic role	[132]
	ORC1	miR-140-5p	Cervical cancer	Oncogenic role	[83]
	Fus	miR-200a		Oncogenic role	[82]
	SIX1	miR-889-3p		Oncogenic role	[84]
	FOXP3	miR-149-3p	Ovarian cancer	Oncogenic role	[85]
	BCL2L2	miR-335		Oncogenic role	[86]
	ANLN	miR-200c-3p	Breast cancer	Doxorubicin resistance	[133]
	GINS2	miR-23a-3p	Melanoma	Oncogenic role	[134]
	MYC	miR-29a	Acute myeloid leukemia	Oncogenic role	[135]
	Bcl-w	miR-497	Extranodal natural killer/T-cell lymphoma	Oncogenic role	[136]
	Smad7	miR-92b	HCC	Tumor suppressor	[87]
	SOX6, PTEN	miR-155-5p		Tumor suppressor	[88]
	PDCD4	miR-497-5p		Tumor suppressor	[89]
	PDK1	miR-139-5p		Oncogenic role	[137]
	ZEB1/2	miR-200b-3p		Oncogenic role	[104]
	O-GlcNAc transferase	miR-424-5p		Oncogenic role	[138]
	PIK3CA	miR-320a		Oncogenic role	[139]
	P21	miR-106b-5p	Renal cell carcinoma	Tumor suppressor	[140]
	CUL3	miR-15a-5p	Acute kidney injury	Pathogenesis promotion	[141]
	PDCD4	miR-142-5p	Acute kidney injury	Pathogenesis promotion	[142]
	YAP	miR-194-5p	Wilms tumor	Oncogenic role	[143]
	CDKN1A	miR-93-5p	Diabetic nephropathy	Promotion of renal interstitial fibrosis	[144]
	TLR4	miR-217	Membranous nephropathy	Promotion of podocyte apoptosis and disease development	[145]
	SOX-6	miR-19b	Renal fibrosis	Apoptosis and inflammation promotion	[146]
	NOD2	miR-320	Atherosclerosis	Promotion of oxidative-LDL-induced cell injury	[147]
	TLR4	miR-370-3p	Pneumonia	Proinflammatory role in LPS-induced injury	[92]
	CCL16	miR-30b-5p	Pneumonia	Proinflammatory role in LPS-induced injury	[148]
	IRF2	miR-204	Respiratory distress syndrome (mice)	Promotion of LPS-induced acute respiratory distress syndrome	[149]
	EGR3	miR-200c-3p	Chronic obstructive pulmonary disease	Apoptosis and inflammation promotion	[150]
	IL-12A	miR-21	Primary graft dysfunction in lung injury	Induction of neutrophil extracellular trap formation and dysfunction progression	[151]
	HMGB3	miR-101-3p	Bronchopulmonar dysplasia	BP dysplasia promotion	[152]
	TLR5	miR-154-5p	Neuropathic pain development (rats)	Neuropathic pain progression	[153]
	SIRT1	miR-30d-5p	Diabetes	Diabetic peripheral neuropathy attenuation	[154]
	Nav1.7	miR-146a	Satellite glial cell activation and inflammatory pain (rats)	Proinflammatory role	[93]
	STAT3	let-7c-5p	Rheumatoid arthritis		[155]
	Smurf1	miR-27a	Microglial cells (spinal cord injury, rats)		[94]
	NFAT5	miR-29c-3p	Epilepsy (rat model)		[156]
	NLRP3	miRNA-223-3p	Renal calculus (mouse model)		[157]
	OPN	miR-376c-5p	Osteoarthritis	Promotion of inflammatory microenvironment and chondrocyte apoptosis	[95]
	CXCR4	miR-211		Promotion of chondrocyte apoptosis	[96]
	MMP-13, ADAMTS5	miR-1277-5p		Promotion of extracellular matrix degradation	[97]
	DNMT3A	miR-149-5p		Promotion of osteoarthritis	[99]
	STAT3	miR-130a		Promotion of inflammation and extracellular matrix degradation	[112]
	SIRT1	miR-653-5p		Protective role	[158]
	ZFPM2	miR-203-3p	Fracture healing	Interferes with proliferation and differentiation of osteoblasts	[159]
	AHNAK	miR-17-5p	Cervical ossification of the Posterior longitudinal ligament	Promotion of osteogenic differentiation	[160]
	PTEN	miR-19	Intervertebral disc degeneration	Autophagy induction	[161]
	BACE1	miR-124	Alzheimer’s disease	Contribution to disease progression	[162]
	Sp1	miR-199a-3p	Parkinson’s disease	Contribution to disease progression	[163]
	BACH1	miR-98	Cerebral injury	Promotion of neuronal injury	[164]
	TIPARP	miR-455-3p		Promotion of neuronal injury	[165]
	FOXO3	miR-27a-3p		Promotion of neuronal injury	[166]
	IKKβ	miR-96-5p		Aggravation of neuronal apoptosis	[167]
	Itga5 or KLF4	miR-92a		Alleviation of cerebral vascular injury	[168]
	COL1A1	miR-29b-3p	Skin fibroblasts (thermal injury)	Promotion of extracellular matrix synthesis, proliferation and migration for wound healing	[169]
	HMGB1	miR-29b	Hepatic stellate cells (alcoholic liver fibrogenesis)	Enhancement ethanol-induced hepatic stellate cell autophagy and activation	[170]
	PDE4D	miR-130a-3p	Myocardial infarction	Promotion of myocardial cell apoptosis and inhibition of cell proliferation	[100]
	Notch1	miR-449	Myocardial infarction	Promotes myocardial cell apoptosis	[101]
	S100B	miR-330-3p	Cardiomyocyte hypertrophy	Antihypertrophy effect	[102]
	TLR2	miR-101	Cardiac hypertrophy	Promotes the progression of cardiac hypertrophy	[103]
	FOXP2	miR-122-5p	Hypoxia-induced H9c2 cardiomyocyte injury	Attenuates hypoxia-induced H9c2 cardiomyocyte injury	[171]
	c-Fos	miR-150-5p	Septic myocardial injury	Induces pyroptosis	[172]
	PTEN	miR-17	Stanford type A aortic dissection (TAAD)	Contribution to disease progression	[173]
	ELN	miR-29b-3p	Thoracic aortic aneurysm	Aggravation of aortic smooth muscle cell apoptosis	[174]
	Arl2	miR-214-3p	Atrial fibrillation	Suppression of myocardial pyroptosis	[175]
FTX	ZEB2,HOXB9, NOB1, YY1	miR-215	Colorectal cancer	Oncogenic role	[176]
	RBPJ	miR-590-5p		Oncogenic role	[177]
	ZFX	miR-144	Gastric cancer	Oncogenic role	[178]
	SIVA1	miR-215			[179]
	AEG-1	miR-342-3p	Glioma	Oncogenic role	[180]
	ALG3	miR-342-3p	Drug resistance in acute myeloid leukemia	Drug resistance	[181]
	c-Met	miR-186	Bone marrow mesenchymal stem cells	Oncogenic role	[182]
	WIF1,PTEN, WNT5A	mir-374a	HCC	Tumor suppressor	[183]
	FOXA2	miR-200a-3p	Lung cancer	Tumor suppressor	[100]
	SOX7	miR-21-5p	Epileptiform hippocampal neurons (rat)	Apoptosis inhibition	[184]
	Bcl2l2	miR-29b-1-5p	Cardiomyocytes (mouse)	Apoptosis inhibition	[185]
	Fmr1	miR-410-3p	Myocardial ischemia/reperfusion injury	Alleviation of hypoxia/reoxygenation-induced cardiomyocyte injury	[186]
JPX	Notch1	miR-137	Osteoclasts (osteoporosis)	Osteogenic differentiation inhibition	[187]
	CCND2	miR-145-5p	Lung cancer	Oncogenic role	[188]
	Twist1	miR-33a-5p		Oncogenic role	[189]
	CXCR6	miR-197	Gastric cancer	Oncogenic role	[190]
	CDH2	miR-944	Oral squamous cell carcinoma	Oncogenic role	[191]
	HIF-1alfa	miR-18a-5p	Intervertebral disc degeneration (human pulposus cells)	Apoptosis inhibition	[192]

### 3.2. ceRNETs Involving FTX

At the time of the preparation of this review, 27 results were retrieved by searching “(FTX) AND (miRNA)” throughout PubMed, and studies matching the criteria reported above were included in Table 1.

The FTX locus produces the lncRNA FTX and multiple intronic miRNAs (Figure 1). It was demonstrated that they are upregulated in colon cancer and cooperatively promote tumor progression. In particular, FTX interacted with DHX9 and Dicer and regulated A-to-I RNA editing and miRNA expression; miR-374b and miR-545 repressed tumor suppressors PTEN and RIG-I to increase proto-oncogenic PI3K-AKT signaling; miR-421 may have an autoregulatory effect on miR-374b and miR-545 [193]. The FTX oncogenic activity in colorectal cancer progression is also mediated by ceRNA activity: FTX binds to miR-215, thus preventing the inhibition of the miRNA targets ZEB2, HOXB9, NOB1 and YY1 related to cell proliferation, migration and invasion; consistently, FTX knock-down suppresses cell proliferation, migration and invasion, and inhibits growth and metastasis in vivo [176]. These results were confirmed by another study performed on colorectal cancer cells, showing the relevance of an additional functional axis, FTX/miR-590-5p/Recombination signal binding protein for the immunoglobulin kappa J region (RBPJ) [177].

Additionally, in gastric cancer FTX exerts a similar functional role via the miR-144/ZFX and miR-215-3p/SIVA1 regulatory axis in promoting tumorigenesis [178,179]; in addition, FTX was upregulated in tumor tissues in comparison to adjacent non-tumor tissues and correlated to poor prognosis [179].

FTX is also endowed with an oncogenic role in glioma, where it is upregulated and promotes cell proliferation and invasion by binding miR-342-3p, resulting in an increased expression of its target AEG-1 [180]. The interaction between FTX and miR-342 was also validated in acute myeloid leukemia cells, resulting in the upregulation of another miRNA target, the ALG3 mannosyltransferase, thus contributing to drug resistance in acute myeloid leukemia.

In contrast with reported role of FTX as a promoter of oncogenesis in different tumors (Table 1), in lung and HCC FTX can work as a tumor suppressor, again via ceRNA activity [183,194]. In particular, in HCC FTX inhibits proliferation and metastasis both in vitro and in vivo by repressing the silencing activity of miR-374a on its targets, i.e., WIF1, PTEN and WNT5A, as negative regulators of the WNT/beta-catenin signaling cascade, that indeed were inhibited, thus promoting cell epithelial–mesenchymal transition and invasion. FTX is expressed at a higher level in female livers compared with male livers; it was found downregulated in HCC tissues, but still maintained at higher levels in females compared with males [183]. It has been suggested that the tumor suppressor role and the reported expression pattern of FTX may contribute to HCC gender disparity, to date attributed solely to sex hormones: males are more susceptible than females to HCC, with average ratios between 2:1 and 4:1 [195,196]; in addition, male HCC patients suffer even worse prognoses than females patients and have shorter survival. Although the notion that estrogen and the estrogen receptor protect women from HCC, thus explaining the rising morbidity in post-menopausal women, and conversely that androgen and androgen receptors confer risk, the role of FTX in HCC, also related to HCC prognosis, and its higher expression in the female liver may contribute to cancer gender difference. Of note, a similar contribution to the HCC gender disparity has been hypothesized for XIST (see above), that is positively regulated by FTX, with an emerging picture that may warrant further investigating, and it may also have implications in gender medicine. Similar considerations could also be applied for glioma and colon and gastric cancer where both XIST and its positive regulator FTX seem to have an oncogenic role (Table 1).

### 3.3. ceRNETs Involving JPX

Ten results were retrieved by searching “(JPX) AND (miRNA)” throughout PubMed; by applying the criteria indicated above, ceRNA activity of JPX was validated for those cases reported in Table 1. Although few works have been published in the field to date, making difficult to speculate/draw conclusions, they demonstrate JPX ceRNET activity, mainly related to cancer.

As XIST, its positive regulator JPX has an oncogenic role in lung cancer, at least partly due to ceRNA mechanisms. First, it is significantly upregulated in NSCLC tissues compared with adjacent normal tissue and in lung cancer metastatic tissues, also correlating to poor survival and malignant phenotypes (tumor size, lymph node metastasis and TNM stage); it promoted cell proliferation in vitro and facilitated tumor growth in a xenograft mouse model [188,189]. Then, it was demonstrated that JPX increased cyclin D2 (CCND2) expression by competitively interacting with miR-145-5p, thus promoting cell proliferation and migration and contributing to cancer progression [188]. Furthermore, JPX also upregulated the transcription factor Twist1 by competitively sponging miR-33a-5p, thus inducing epithelial–mesenchymal transition (EMT) and cancer cell invasion [189]. Both ceRNA networks ultimately contribute to regulating lung tumorigenesis and metastasis, indicating potential therapeutic targets and novel biomarkers.

An oncogenic potential has also been verified in oral squamous cell carcinoma, where JPX contributes to cell proliferation, migration and invasion via the miR-944/CDH2 axis [191].

Finally, similarly to that reported for XIST and the other positive regulator FTX, JPX has an oncogenic role in gastric cancer and again the underlying molecular mechanism is based on a ceRNA mechanism: JPX increased the chemokine receptor CXCR6 by sponging its inhibitory miR-197 and thus promoting gastric cancer progression [190].

## 4. Implications for X Chromosome Aneuploidy Syndromes

In the scenario of ceRNA activity, miRNA binding sites can be envisioned as “the letters of an RNA code” by which transcripts communicate and regulate their relative expression levels [14]. Indeed, a transcript from a pseudogene can also regulate expression of its related gene by competitively binding shared miRNAs [18]. Genetic events such as copy number variation (amplification or loss) could induce “coding-independent” effects by altering the levels of microRNAs available for silencing particular transcripts. In this view, it should be noted that the X chromosome has the highest density of miRNA sequences compared to the other chromosomes, an evolutionarily conserved mammalian feature that equips females with a larger miRNA regulatory machinery than males [197,198]. Different syndromes related to X chromosome aneuploidies have been reported, characterized by either gain or loss of the entire chromosome (aneuploidy), or parts of the chromosome (structural abnormalities, e.g., isochromosomes) [199]. In particular, the partial or complete lack of a second X chromosome causes Turner syndrome (TS), estimated 1 in 2000 females. Forty to fifty percent of TS women have the 45,X karyotype, whereas the remaining cases are represented by mosaicism (mainly 45,X/46,XX mosaicism), where 20% of cases have alterations of the X chromosome (isochromosome Xq or ring X chromosome) and 10–12% of cases have differing amounts of Y chromosome material. Phenotypic traits include short stature, ovarian insufficiency and infertility, cardiac malformations, autoimmune diseases, metabolic disorders, neurocognitive problems [200]. The presence of an additional X chromosome in males causes Klinefelter syndrome (KS), with an incidence of 1 in every 660 male births. KS is characterized by a 47,XXY karyotype in about 80–90% of affected men, whereas the remaining cases are represented by mosaicism (usually 47,XXY/46,XY), higher grade sexual chromosome aneuploidies (e.g., 48,XXYY; 48,XXXY; 49,XXXXY) or X chromosome structural abnormalities. Phenotypic traits include tall stature, hypergonadotropic hypogonadism, infertility, type 2 diabetes, autoimmune disorders, neurocognitive problems, some cardiovascular abnormalities, obesity [201]. An additional X chromosome in females causes triple X syndrome (47,XXX), with an incidence of 1 in 1000 live-born girls. Phenotypic traits include a lower IQ, an increased incidence of psychiatric, language and autism-like disorders and premature ovarian failure [202]. In those syndromes, no obvious genotype–phenotype relationship has been established to date, besides the importance of the SHOX gene situated in the pseudo-autosomal region and therefore expressed on all sex chromosomes because it does not undergo X inactivation, and which has been shown to impact height and other skeletal changes, especially seen in patients with Turner syndrome [203,204], and explains part of the short stature in Turner syndrome and increased height in conditions with supernumerary sex chromosomes [205]. Patients with sex chromosome aneuploidies carrying the same karyotype can exhibit quite different traits and a range of comorbidities, suggesting a role of epigenetic mechanisms behind sex chromosome aneuploidy [206,207]. The absence or excess of escape X-linked miRNAs and other RNA species could greatly contribute to the observed variability in clinical traits, and it may be speculated that some of the ceRNETs discussed above could also be involved. Intriguingly, an increased expression of the lncRNAs XIST, JPX and TSIX was observed by comparing X chromosome RNA expression of blood cells in TS, female controls and triple X syndrome [208], as also found by others [209]. Moreover, the differential exon usage observed in the same study for several genes may have a functional significance in terms of ceRNA activity, beyond the coding potential, since they carry different binding sites for miRNAs.

Exploring ceRNET hypotheses in the context of X aneuploidy syndromes could contribute to filling the gap between puzzling clinical data and underlying molecular mechanisms, beyond a simple karyotype, also disclosing new perspectives in terms of innovative diagnostics tools and development of more effective therapeutic/patient-management strategies. As an example, higher expression of XIST has been linked to breast cancer pathogenesis and chemoresistance (Table 1) [133,210] and here the very low expression of XIST could at least partly underlie the very low risk of breast cancer among TS women and, vice versa, the higher XIST expression in KS men involved in the increased breast cancer incidence [211,212], which to date has not been explained convincingly by other data.

## 5. Conclusions

It has become increasingly clear that RNA molecules, either coding or non-coding, perform a variety of functions previously unexplored and their crosstalk provides regulatory networks governing numerous biological processes and whose derailing has pathological consequences, such as cancer.

**Figure 2:**
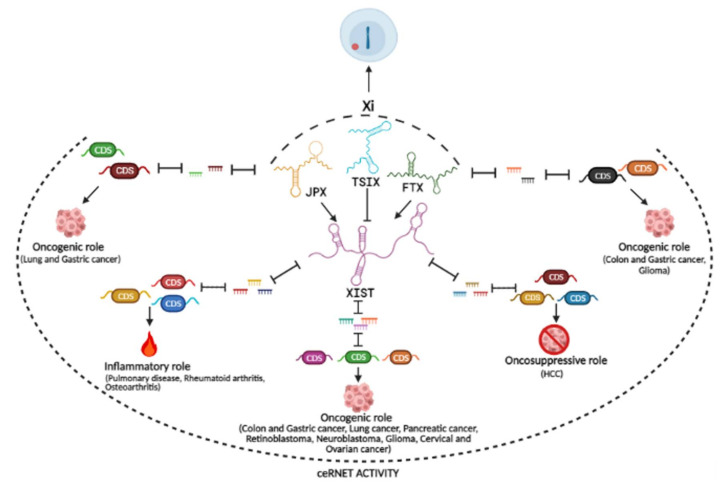
Multifaceted role of lncRNAs from the XIC. XIST is the essential molecule for the X chromosome inactivation (Xi); its ceRNA activity is invoked in different pathways, such as those indicated, with specific examples in brackets. JPX, FTX and TSIX are other XIC lncRNAs acting as positive or negative regulators of XIST, thus contributing to Xi; they are also endowed with ceRNET activity, to date less well characterized. Figure created with BioRender.com.

Many years after its discovery, XIST, and other lncRNAs from the XIC, unveil their regulatory potential beyond their role in dosage compensation (Figure 2). While intriguing and somehow pioneering, the reported studies clearly demonstrate the ceRNET activity of some lncRNAs from the XIC, often focusing on the interaction of a single lncRNA with one miRNA shared with one competing target transcript; however, the possible high number of miRNA binding sites on the >19 kbp mature RNA such as XIST suggests a more complex potential landscape of shared miRNAs and their targets. Probably, the definition of such large-scale regulatory networks across the transcriptome will represent the next challenge. Moreover, no data have yet been published for many pseudogenes from the XIC (Figure 1), often overlapping with the main transcripts; their investigation and possible competing interactions with shared miRNAs may provide interesting findings, not only for “basic research”, but also for clinical perspectives. In fact, growing evidence linking pathological conditions to lncRNAs and their regulatory network is emerging, especially in cancer development and progression, with important implications in diagnostics and therapeutics. Specifically, different studies report the correlation of XIST expression with prognosis such as in the case of colon and gastric cancer, glioma and cervical and ovarian cancer [65,66,67,68,69,76,77,78,79,83,84,85,86] or FTX expression and gastric cancer prognosis [179] or JPX expression and poor survival of lung cancer patients [188,189]; probably, combination of the expression values of more than one RNA molecule involved in the same regulatory axis could enhance the diagnostic power, e.g., making it possible to stratify the patients according tumor stage, favorable/unfavorable outcome and even therapy effectiveness. Finally, understanding the versatility of RNA biology has become a stepping stone for RNA therapeutics that has definitely demonstrated its power [13]. Various therapeutic strategies can be based on boosting the expression level of a “beneficial” miRNA or inhibiting a harmful one; of note, different studies demonstrated that knock-down of a lncRNA such as XIST [68,69,75,76,77,78,79,83,84,85,86], or FTX [176] and JPX [188,189], exerts tumor-suppressive functions in vitro and in vivo, validating new therapeutic targets. In the near future, some of the predicted RNA interactions among lncRNAs/miRNAs/mRNAs that have not been included here could be experimentally validated, especially by in vivo studies, allowing a comprehensive view and envisaging new therapeutic opportunities by manipulating the network. Finally, the ceRNET activity of lncRNAs from the XIC highlighted in this review may suggest the importance of also considering the gender context in non-coding RNA studies, to fill the gap between clinical data and the understanding of molecular mechanisms underlying some sex-biased diseases, such as different tumor susceptibility (e.g., HCC).

The research field involving large-scale RNA regulatory networks is still in its infancy, and requires studies able to integrate computational analyses and new experimental platforms to fulfill the promise of significant advancement in the molecular knowledge of biological systems, and implications in sex-specific genome regulation and gender medicine.
